# Experimental verification of SO_2_ and S desorption contributing to defect formation in MoS_2_ by thermal desorption spectroscopy†

**DOI:** 10.1039/d2na00636g

**Published:** 2022-11-28

**Authors:** Shuhong Li, Tomonori Nishimura, Mina Maruyama, Susumu Okada, Kosuke Nagashio

**Affiliations:** a Department of Materials Engineering, University of Tokyo Tokyo 113-8656 Japan nagashio@material.t.u-tokyo.ac.jp; b Department of Physics, University of Tsukuba Tsukuba Ibaraki 305-8577 Japan

## Abstract

The defect-free surface of MoS_2_ is of high importance for applications in electronic devices. Theoretical calculations have predicted that oxidative etching could be responsible for sulfur vacancy formation. No direct experimental evidence, however, points out the role of adsorbed oxygen on sulfur vacancy formation for MoS_2_, especially on an insulating SiO_2_/Si substrate. Herein, by applying thermal desorption spectroscopy, we found that sulfur loss can be tightly coupled to adsorbed oxygen, as confirmed by observation of SO_2_ desorption. With annealing MoS_2_, even under ultrahigh vacuum, oxygen molecules adsorbed on MoS_2_ assist the sulfur atom in dissociating from MoS_2_, and thus, defects are formed as the result of SO_2_ desorption from 200 °C to 600 °C. At higher temperatures (over 800 °C), on the other hand, direct sulfur desorption becomes dominant. This finding can be well explained by combining the morphology investigation enabled by atomic layer deposition at defective sites and optical transitions observed by photoluminescence measurements. Moreover, a preannealing treatment prior to exfoliation was found to be an effective method to remove the adsorbed oxygen, thus preventing defect formation.

## Abstract 2

We confirm that adsorption of oxygen results in the formation of defects by detecting SO2 desorption though thermal desorption spectroscopy.

## Graphical abstract

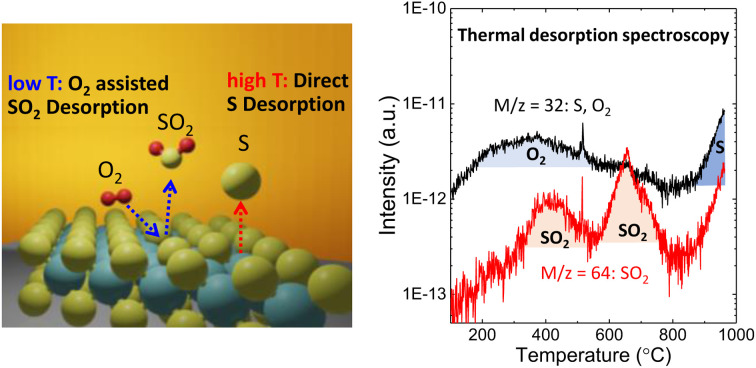

## 1. Introduction

MoS_2_, a two-dimensional (2D) layered material, is promising as a channel material for next-generation field-effect transistors (FETs) because a natural thin body can overcome the scaling limit for the Si gate length.^1,2^ Although the dangling-bond-free surface of the layered MoS_2_ channel is expected to ideally provide an electrically inert interface, sulfur vacancies in reality have been recognized as a dominant defect in MoS_2_ due to the lowest formation energy of ∼1.3–1.5 eV under S-poor conditions.^3,4^ Moreover, sulfur vacancies are known to introduce defect states in the band gap, which degrades the FET performance.^5–8^ Therefore, controlling sulfur vacancies has still been an critical issue due to the limited understanding of its formation mechanism, even though many healing processes based on various adsorbates or S vapor annealing have been proposed.^9–12^

The stability of MoS_2_ has been intensively studied thus far.^13,14^ The conventional mechanical exfoliation process for device fabrication leads to exposure to ambient air, which introduces adsorbates on the MoS_2_ surface and edge. These adsorbates, such as oxygen and water, greatly affect chemical stability, since thermodynamic calculations suggest that most 2D materials show oxidation tendencies.^15^ The long-term exposure of MoS_2_ flakes to ambient air has proven the gradual oxidation from the edges to the interior of MoS_2_ ^16^ because the coordinatively unsaturated edge is energetically more favorable for oxidation than the basal plane.^17–20^ For intentional exposure to an oxygen environment,^21^ the basal plane of MoS_2_ is oxidized, and MoO_3_ is formed during oxidation above 400 °C. This is further supported by the aggressive oxidation using oxygen plasma, where layer-by-layer oxidation was observed since MoO_3_ formed on the MoS_2_ surface prevented further oxidation.^22^ Interestingly, for an oxygen/air environment at lower temperatures of 300–340 °C, however, layer-by-layer anisotropic etching of MoS_2_ results in triangular pits, which are initiated *via* intrinsic defects on the basal plane of MoS_2_.^21,23^ This oxidative etching is explained by the reaction of MoS_2_ + O_2_ → MoO_3_↑ + SO_2_↑,^18,24^ where both MoO_3_ and SO_2_ are volatile. Thus, oxidative etching is considered to be an important process when MoS_2_ is exposed to oxygen or air.

Recently, atomic-resolution scanning tunneling microscopy (STM) suggested that the O_2_ adsorbed on the basal plane of MoS_2_ volatized as SO_2_ by removing S, leaving S vacancies with O saturation on the basal plane.^10^ This O_2_-assisted S vacancy formation in ambient air at room temperature (RT) is estimated to be energetically spontaneous with −0.49 eV by first principles calculation. On the other hand, in a high vacuum environment, the creation of S vacancies has been confirmed not at 127 °C but at elevated temperatures >627 °C by atomic-resolution STM^25^ and transmission electron microscopy (TEM).^26^ These results indicate that the formation of S vacancies at RT cannot be fully explained without the existence of oxygen adsorbed on the basal plane of MoS_2_, suggesting the importance of O_2_ adsorption and reaction mechanism. Therefore, further theoretical studies on the initial step of oxidative etching have been conducted in detail.^27^ However, direct experimental evidence of SO_2_ desorption as well as direct S desorption has not yet been proven.

Here, thermal desorption mass spectrometry (TDS) is used to study the surface reaction and desorption kinetics in Si^28,29^ Ge,^30^ and HOPG^31^ by detecting a small amount of desorption species using a quadrupole mass spectrometer (QMS). By using this TDS system, it may be possible to detect SO_2_ and/or S desorption from MoS_2_ flakes transferred onto SiO_2_/Si substrates in ambient air. Although no measurement method to observe S vacancies with atomic resolution is available for MoS_2_ on an insulating substrate, it is well known that Al_2_O_3_ during atomic layer deposition (ALD) is adsorbed only at the defect sites;^32–35^ this will allow us to visualize the defect evolution with the assistance of surface topology observed macroscopically by atomic force microscopy (AFM). In this research, a quantitative characterization of the defect evolution mechanism of MoS_2_ on SiO_2_ at elevated temperatures up to ∼1000 °C is realized by combining TDS and ALD.

## 2. Experimental

**Fig. 1:**
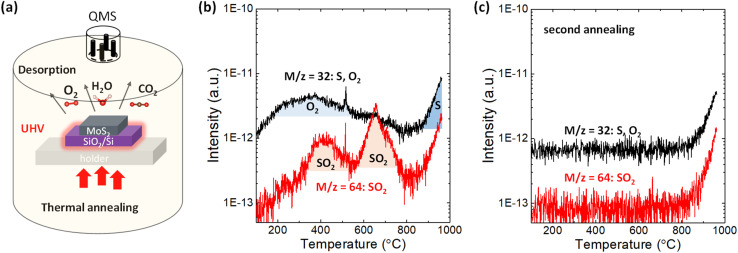
(a) Illustration of thermal desorption spectroscopy applied in this experiment. (b) TDS spectra with *m*/*z* = 32 (O_2_ or S) and *m*/*z* = 64 (SO_2_) for bulk MoS_2_ flakes on the SiO_2_/Si substrate. (c) Comparison of the TDS spectra for *m*/*z* = 32 between the first annealing and second annealing.

2H-MoS_2_ crystals purchased from SPI supplies were used in this study. First, a 110 nm SiO_2_/*n*^+^-Si substrate was sonicated by acetone and isopropyl alcohol for 10 min in laboratory air. After a few exfoliations by metallic tweezers, the bulk MoS_2_ flakes were directly transferred to the SiO_2_/*n*^+^-Si substrate. This procedure provides a sufficiently large surface area of MoS_2_ for the TDS measurements, while it prevents any tape residue on the substrate since the tape is not used. Other 2H-bulk transition metal dichalcogenides (WS_2_, WSe_2_, MoSe_2_ and MoTe_2_) were grown by chemical vapor transport method^36^ and treated by the same method applied for MoS_2_. Alternatively, MoS_2_ flakes were clashed into powders using a mortar to further increase the surface area of MoS_2_. Then, the MoS_2_ powders were placed on the SiO_2_/Si substrate. Fig. 1a illustrates the TDS apparatus, which allows selective heating of only the Si substrate *via* infrared radiation from the underlying power-operated lamp under ultrahigh vacuum (UHV) conditions of ∼4 × 10^−8^ Pa. Since this TDS is cold wall system, the desorption from the inner chamber wall can be avoided during the sample heating. The QMS functions by manipulating the electric field between four orthogonal rods to collect ionized desorbed molecules and atoms and thus enables distinguishing the desorbed species according to its atomic/molecular mass-to-charge ratio. The bulk MoS_2_ flakes were heated from room temperature to ∼1000 °C with a heating rate of 20 °C min^−1^, and TDS spectra were detected in multi-ion detection mode with a sensitivity up to 10^−15^ A.

For ALD, Al_2_O_3_ was deposited on MoS_2_ flakes in a hot wall chamber at 200 °C with trimethylaluminum (TMA), water, and nitrogen as the precursor, oxidant, and purge gas, respectively.^32^ The pulse time for TMA/water is 0.1 s/0.5 s, respectively. Ten cycles were performed to obtain ∼2 nm-thick Al_2_O_3_. The surface morphology was measured with dynamic force mode by AFM. The Raman and photoluminescence (PL) spectra were measured at RT in ambient air using a 488 nm excitation laser with a power of 0.06 mW to avoid the degradation of MoS_2_ owing to laser heating.

## 3. Results and discussion

First, the desorption of adsorbates on the SiO_2_/Si substrate without MoS_2_ flakes should be examined. As shown in Fig. S1a,† in addition to the small amount of H_2_, CO_2_ and N_2_, H_2_O physiosorbed on the SiO_2_/Si substrate was clearly detected in the temperature range of 100–300 °C because the SiO_2_ surface is hydrophilic.^37^ After transferring MoS_2_ flakes onto the SiO_2_/Si substrate, the desorption of H_2_O and CO_2_ was enhanced, as shown in Fig. S1b.† This indicates that many kinds of gas species are adsorbed on MoS_2_ flakes when MoS_2_ flakes are mechanically transferred in ambient air.

The desorption of sulfur and sulfur-related compounds is of the highest interest. Fig. 1b shows the TDS spectra for mass-to-charge ratios of *m*/*z* = 32 and 64. S and O_2_ can be assigned for the same mass-to-charge ratio of *m*/*z* = 32 because QMS can only filter substances by mass-to-charge ratio. To separate them, the second annealing experiments were successively carried out without removing the sample, as shown in Fig. 1c and S1c.† The broad peak from 200 to 600 °C for *m*/*z* = 32 is totally removed in the second annealing experiment, with the sharp intensity tail remaining at ∼800 °C. It can be expected that direct S desorption is observable even in the second annealing, while O_2_ is not detected once it has totally desorbed in the first annealing experiment. Therefore, the broad peak from 200 to 600 °C in Fig. 1b is assigned as the contribution from O_2_. Here, it should be noted that the contribution from water adsorbed to the sample was excluded in this discussion, even though the water desorption was observed in Fig. S1.† According to the recent paper,^38^ no degradation of monolayer MoS_2_ was observed after two weeks exposure to the environment of both N_2_ and N_2_ with the 75% humidity, suggesting that water itself have no dominant contribution for oxidation. Moreover, the desorption tails observed for all species at a high temperature of ∼800 °C in Fig. S1† basically resulted from the inner pressure enhancement in the TDS chamber, not from the real increase in desorption. Nevertheless, the TDS spectrum of *m*/*z* = 32 in the second annealing experiment is greatly enhanced when the surface area of MoS_2_ increases by preparing the MoS_2_ powder, which is reflected as a threshold temperature shift to the lower side in Fig. S2a.† This indicates that the desorption tail for *m*/*z* = 32 contains real desorption, that is, the contribution from S. Moreover, Fig. S2b† compares the TDS spectra of chalcogens (S, Se and Te) for various transition metal dichalcogenides. The direct Te (*m*/*z* = 128) desorption from MoTe_2_ is stronger than others, which is consistent with the common understanding of the stability of 2D materials.

In addition to direct S desorption, the sulfur oxidation product, that is, SO_2_, was also observed and expressed as *m*/*z* = 64 in Fig. 1b, where two peaks are observed at ∼400 °C and ∼650 °C. It should be noted that the *m*/*z* = 64 was assigned to be the SO_2_ rather than S_2_ based on the isotope method.^39^ Sulfur mainly has stable four isotopes: ^32^S (95.02%), ^33^S (0.5%), ^34^S (4.21%), and ^36^S (0.02%). The second abundant ^34^S (*m* = 34) was used to distinguish S_2_ (*m*/*z* = 68) and SO_2_ (*m*/*z* = 66), as shown in Fig. S3(b).† The desorption signal of *m*/*z* = 66 well follows *m*/*z* = 64, while no clear feature was observed for *m*/*z* = 68. Moreover, from the viewpoint of activation energy of S_2_ desorption, the higher temperature is expected. Therefore, *m*/*z* = 64 was assigned to SO_2_ rather than S_2_. Furthermore, in the second annealing experiment in Fig. 1c, both peaks disappeared, suggesting that oxygen that constitutes SO_2_ comes from adsorbates on the MoS_2_ flakes not from inside of the MoS_2_ flakes. Therefore, it is considered that adsorbed O_2_ reacted with S in MoS_2_ and desorbed as SO_2_ at ∼400 °C and ∼650 °C. That is, it is suggested that sulfur vacancy formation at relatively low temperature (∼400 °C & 650 °C) is caused by SO_2_ desorption assisted by oxygen adsorption on the MoS_2_ basal plane, while it is caused by direct S desorption at high temperature (over 800 °C). Although the theoretical simulation^10^ has suggested SO_2_ desorption instead of direct S desorption for S vacancy formation, this is the first experimental observation.

The origin of the two SO_2_ peaks in Fig. 1b may be related to different intermediates for the final SO_2_ formation, as suggested in the theoretical calculation.^27^ The formation of the intermediate “OSOMo”, in which one oxygen atom is inserted into the Mo–S bond, leads to the desorption of SO_2_ with an energy barrier of 1.49 eV. On the other hand, the formation of the intermediate “Mo–OSO”, which can be seen as the SO_2_ molecule with one oxygen atom bonded to two adjacent Mo atoms, leads to the desorption of SO and SO_2_ with energy barriers of 0.41 eV and 0.78 eV, respectively. Interestingly, in the reproduced TDS experiment in Fig. S3,† it is found that the SO signal highly overlaps with the SO_2_ signal in the low-temperature region, where the first peak is observed in Fig. 1b. The overlapping feature suggests that the formation of SO and SO_2_ at relatively low temperatures is simultaneous. The slight intense peak for SO at 650 °C can be attributed to the decomposition of SO_2_ to SO and O due to the ionization in QMS.^39^ Although the rigorous separation of SO and SO_2_ is difficult at present, two SO_2_ peaks may result from the different intermediates for the final SO_2_ desorption.

**Fig. 2:**
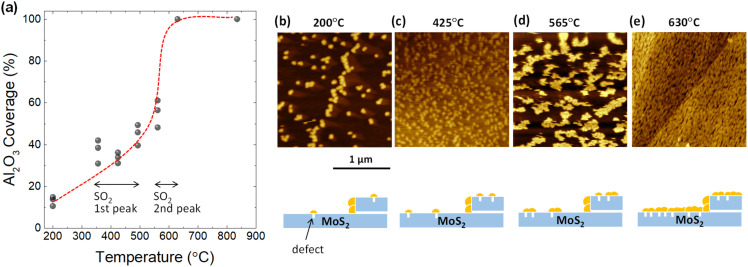
(a) Coverage estimation of ALD-Al_2_O_3_ on TDS-annealed bulk MoS_2_ flakes with varying annealing temperatures. The gray point represents the coverage extracted from different AFM images. The red dashed line is an eye guide to reflect the coverage evolution rate. (b)–(e) Representative AFM images for Al_2_O_3_/MoS_2_ at annealing temperatures of 200 °C, 425 °C, 565 °C, and 630 °C, respectively. In addition, the Al_2_O_3_ coverage evolution with increasing annealing temperature is schematically illustrated. The yellow particles represent deposited Al_2_O_3_.

To confirm whether defects form as a result of desorption during TDS annealing, 2 nm Al_2_O_3_ was deposited by ALD since Al_2_O_3_ is formed selectively on defect sites due to the dangling-bond free inert surface of the 2D material.^32–35^ For the present ALD-assisted morphology characterization, bulk MoS_2_ were intentionally selected to avoid strain-enhanced ALD growth on monolayer MoS_2_ due to the transfer process. To show the importance of ALD, the surface topography of MoS_2_ annealed up to 835 °C in the TDS chamber without ALD was examined to assess the resolution of AFM. As shown in Fig. S4,† no clear defects could be identified. The situation was inverted when ALD was applied to the MoS_2_ flake after TDS annealing. As shown in Fig. 2b, for the MoS_2_ flake annealed up to 200 °C in the TDS chamber, the growth of Al_2_O_3_ is distributed in dots on the MoS_2_ surface with a greater concentration at the step edge. It should be noted that 200 °C was selected as the starting temperature for defect characterization since the Al_2_O_3_ growth temperature in ALD was 200 °C. As the TDS annealing temperature increases, Al_2_O_3_ growth is enhanced, occupying more surface area of MoS_2_ (Fig. 2b–e). For the MoS_2_ flake annealed up to 630 °C, the growth of Al_2_O_3_ is no longer preferential at the grain boundaries and the step edges but uniformly covers the entire MoS_2_ surface. The morphology evolution is schematically illustrated below the AFM images.

Next, the Al_2_O_3_ coverage was extracted from the AFM images and plotted as a function of annealing temperature in Fig. 2a. The coverage rate is relatively slow in the temperature range from 350 to 500 °C, where the first peak of SO_2_ desorption is observed in Fig. 1b. In contrast, there was a significant increase in the coverage rate from 550 to 630 °C. This temperature interval matches the second peak of SO_2_ desorption. These results suggest that the evolution of the Al_2_O_3_ coverage is correlated to SO_2_ desorption. However, further quantitative estimation of the defect density is not possible here since the Al_2_O_3_ particle density does not form a one-to-one correspondence with the defect density. That is, new Al_2_O_3_ growth occurs not only at the defect site but also on the Al_2_O_3_ that has already been grown. However, the importance of ALD-assisted AFM characterization is to provide clear evidence that the formation of defects on the basal plane of MoS_2_ flakes does arise when SO_2_ desorption occurs.

**Fig. 3:**
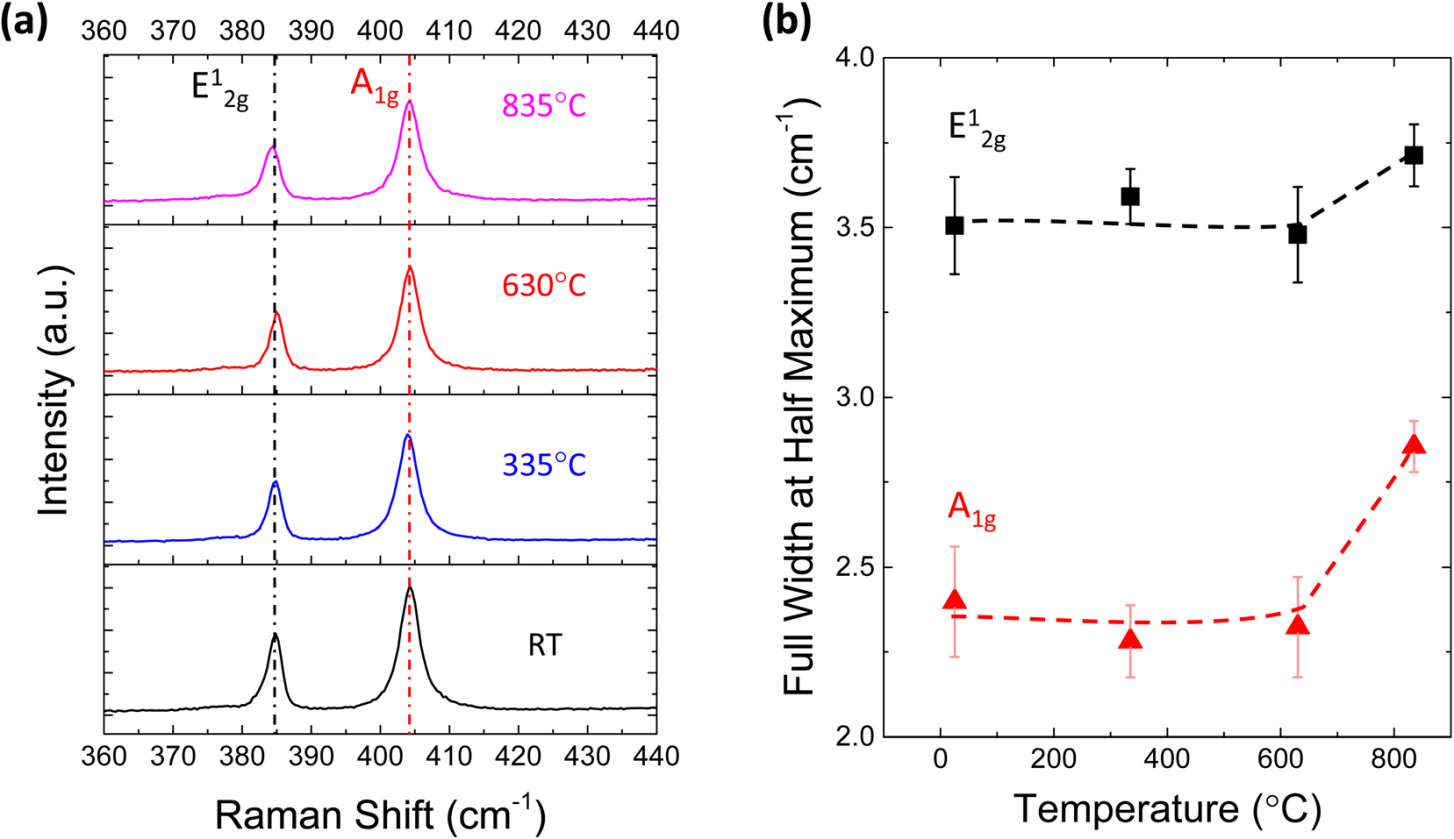
(a) Raman spectra for monolayer MoS_2_ annealed at different temperatures in the TDS chamber. (b) Full width at half maximum (FWHM) of the Raman peaks at different temperatures.

To reveal the structural change in MoS_2_ after TDS annealing, Raman measurements were conducted. Fig. 3a shows the Raman spectra of monolayer MoS_2_ at different TDS annealing temperatures. The frequency difference of E^1^_2g_ and A_1g_ is ∼19.4 cm^−1^, which matches well with the characteristics of monolayer MoS_2_.^40^ No detectable shifts of the E^1^_2g_ and A_1g_ peaks were found for any temperature range. On the other hand, the full width at half maximum (FWHM) of these two peaks shown in Fig. 3b is slightly broadened when the annealing temperature was elevated to 835 °C; at this temperature, direct S desorption becomes dominant in the TDS spectra of Fig. 1b. This indicates that some damage to the crystallinity is induced by considerable direct S desorption. The change in Raman spectra at temperatures above 800 °C has also been reported.^41^ Although Raman spectra are found to be insensitive to temperatures below 630 °C, this is not consistent with Fig. 2a, where a clear Al_2_O_3_ coverage evolution is observed. In Fig. S3,† the desorption of SO_2_ and S was detected but not for MoO, MoO_2_, and MoO_3_, suggesting that the original MoS_2_ crystal lattice can be retained since Mo is located at the center of the MoS_2_ crystal structure. Therefore, Raman spectra could be insensitive to the relatively small change in crystallinity due to S vacancy formation accompanied by SO_2_ desorption.

**Fig. 4:**
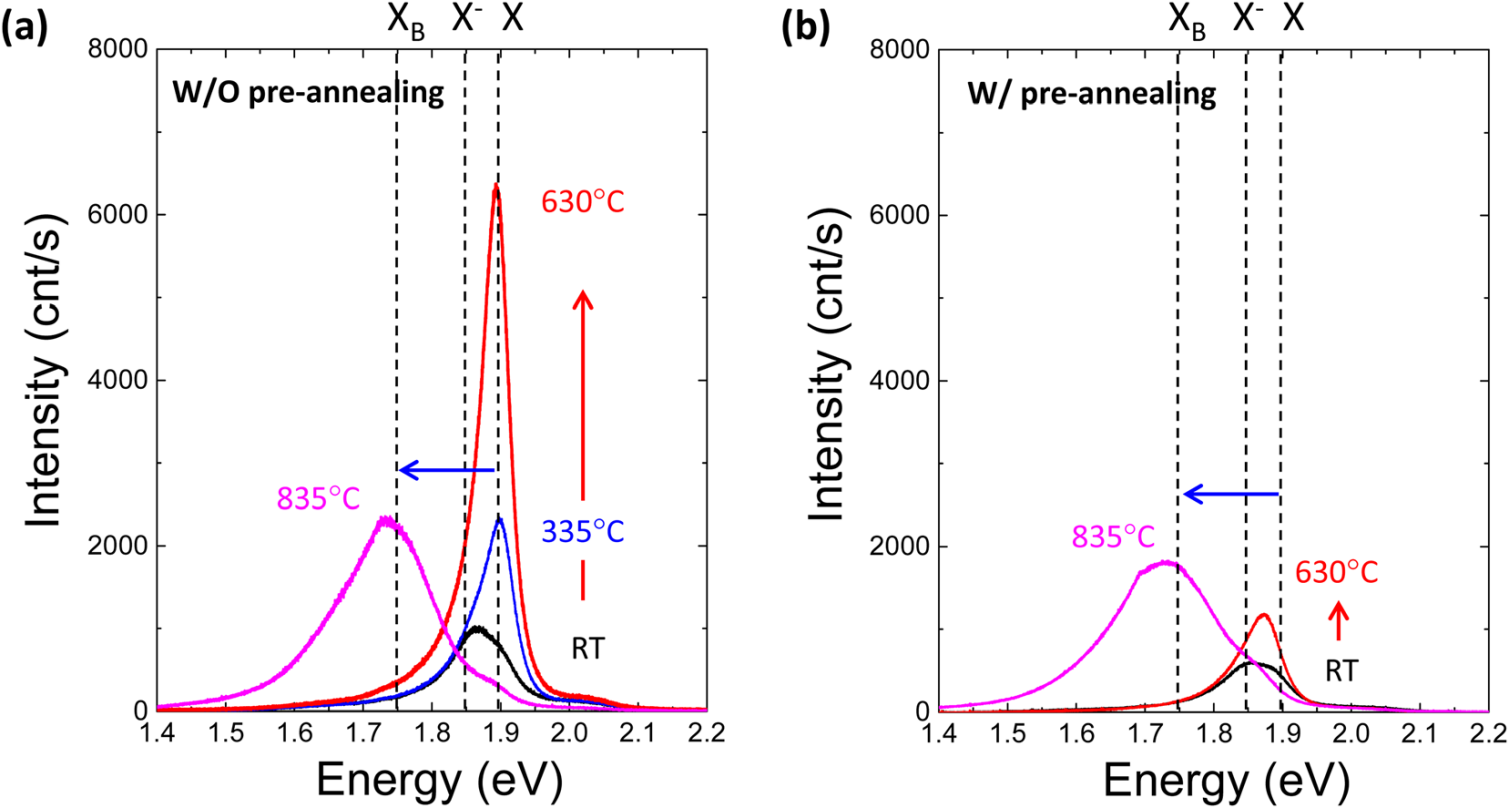
(a) Photoluminescence spectra for monolayer MoS_2_ annealed at different temperatures in the TDS chamber without preannealing in Ar. X, X^−^ and X_B_ represent exciton, trion and defect-induced bound exciton, respectively. The red arrow indicates the intensity enhancement with increasing annealing temperature from RT to 630 °C, while the blue arrow indicates the transition from exciton to defect-induced bound exciton due to high-temperature annealing at 835 °C. (b) Photoluminescence spectra for monolayer MoS_2_ annealed at different temperatures in the TDS chamber with preannealing in Ar.

To further investigate the effect of defect formation more sensitively, PL measurements were performed for monolayer MoS_2_ annealed at different temperatures in the TDS chamber. As shown in Fig. 4a, the conventional broad PL peak mixed with exciton (X, ∼1.9 eV) and trion (X^−^, ∼1.85 eV) was observed.^42–44^ As the TDS annealing temperature increased from RT to 630 °C, the intensity of the exciton peak drastically increased. Since the MoS_2_ flakes were exposed to ambient air during the PL measurement after removing the sample from the TDS chamber, the exciton intensity enhancement is attributed to the oxygen chemical adsorption on S vacancies created by SO_2_ desorption during TDS annealing.^43^ As shown in Fig. S5,† the trion contribution was reduced at elevated temperature, while the exciton component became dominant. This indicates the p-type doping by oxygen passivation at the defect site.^45^ Moreover, when the annealing temperature increased to 835 °C, the defect-induced bound exciton peak (X_B_, ∼1.75 eV) became dominant.^46–49^ Although SO_2_ formation at relatively low temperatures is limited by the amount of oxygen adsorbed initially on MoS_2_ flakes, direct S desorption at high temperatures depends only on the temperature. Therefore, a much broader X_B_ peak implies a significant amount of S vacancy formation and clustering due to the enhanced direct S desorption, which is also supported by the broadening of the FWHM in the Raman peaks (Fig. 3b).

Four different types of experiments, namely, of TDS, AFM, Raman and PL, surprisingly, can be well explained by the two common kinds of defect formation mechanisms in the low and high annealing temperature regions. That is, at a low annealing temperature, the oxygen adsorbed on the basal plane of MoS_2_ flakes is the key factor, and it volatizes as SO_2_ by removing S from MoS_2_, leaving S vacancies. Then, at a high annealing temperature, direct S desorption becomes dominant. Here, it is reasonable to consider that defect formation at low annealing temperatures could be suppressed by removing oxygen adsorbed on MoS_2_ flakes before TDS annealing. To verify this idea, the MoS_2_ crystals were preannealed at 500 °C for one hour in an Ar gas flow to remove oxygen adsorbed on the MoS_2_ surface. Then, MoS_2_ flakes were transferred onto the SiO_2_/Si substrate by mechanical exfoliation in ambient air, followed by TDS annealing. The PL spectra of preannealed monolayer MoS_2_ are shown in Fig. 4b. The PL intensity enhancement at temperatures below 630 °C is greatly suppressed, which strongly indicates that S vacancy formation due to SO_2_ desorption was hindered due to the great reduction in oxygen adsorbed on the MoS_2_ surface. On the other hand, at a high temperature of 835 °C, the X_B_ peak was almost the same, which is also evidence for direct S desorption. These results clearly support the two kinds of defect formation mechanisms in the low and high annealing temperature regions. To date, S vacancies have been experimentally observed and recognized as dominant defects because they have the lowest formation energy of ∼1.3–1.5 eV compared with other types of defects.^3,4^ However, this large formation energy could not explain the high S vacancy concentration of ∼10^13^ cm^−2^.^50^ The present study experimentally proves that oxygen adsorbed on MoS_2_ assists S vacancy formation by desorbing as SO_2_ since this O_2_-assisted S vacancy formation is energetically spontaneous at −0.49 eV.^10^

## 4. Conclusions

Through this experimental desorption study using TDS in combination with ALD, Raman, and PL, two kinds of S vacancy formation mechanisms are realized, that is, O_2_-assisted SO_2_ desorption at low annealing temperatures (400 °C to 650 °C) and direct S desorption at high annealing temperatures (over 800 °C). The key finding is that the initially adsorbed oxygen causes S vacancy formation through SO_2_ desorption. As a solution, the removal of oxygen is quite effective for preventing the formation of defects and further oxidation.

## Conflicts of interest

There are no conflicts to declare.

## Supplementary Material

NA-005-D2NA00636G-s001
