# Supplementary material for: Experimental verification of SO2 and S desorption contributing to defect formation in MoS2 by thermal desorption spectroscopy
Source: Nanoscale Adv. 2022 Nov 28;5(2):405–11. doi: 10.1039/d2na00636g (PMC9846482; doi:10.1039/d2na00636g)
Supplement: NA-005-D2NA00636G-s001 [file NA-005-D2NA00636G-s001.pdf]

Electronic Supplementary Material (ESI) for Nanoscale Advances.
This journal is © The Royal Society of Chemistry 2022

Supplementary information

Experimental verification of SO_2 and S desorption contributing to defect formation in MoS_2 by thermal desorption spectroscopy

Shuhong Li,[a,b] Tomonori Nishimura,[a] Mina Maruyama,[b] Susumu Okada [b] and Kosuke Nagashio*[a]

[a]*Department of Materials Engineering, University of Tokyo, Tokyo 113-8656, Japan*

[b]*Department of Physics, University of Tsukuba, Tsukuba, Ibaraki, 305-8577 Japan*

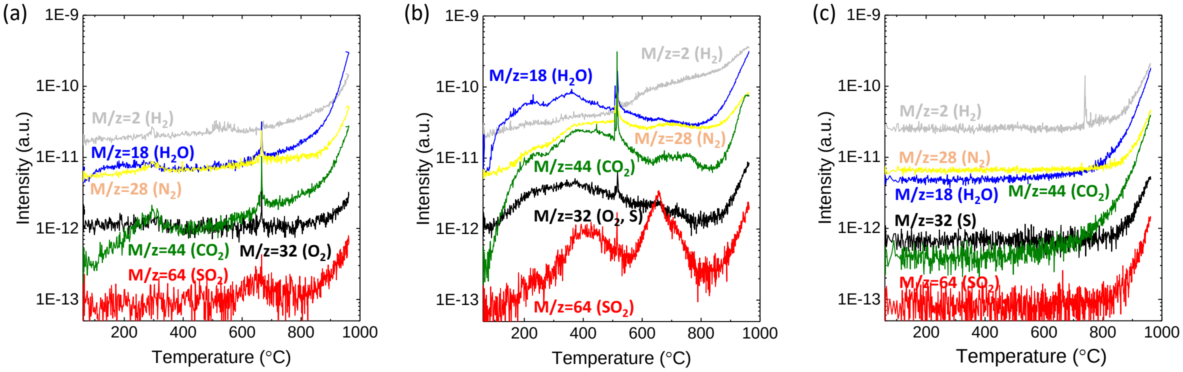

Fig. S1 (a) TDS spectra for the main desorbed species detected from the SiO_2/Si substrate. The spikes at approximately 500 °C result from the fluctuation of the inner pressure in the TDS chamber. (b) TDS spectra for the main desorbed species detected from the MoS_2 flakes on the SiO_2/Si substrate. (c) The second annealing TDS spectra for the MoS_2 flakes on the SiO_2/Si substrate. The removal of adsorbed species on the MoS_2 flakes can be reflected by the flatness of the signal before increasing the inner pressure in the high-temperature region (over 800 °C).

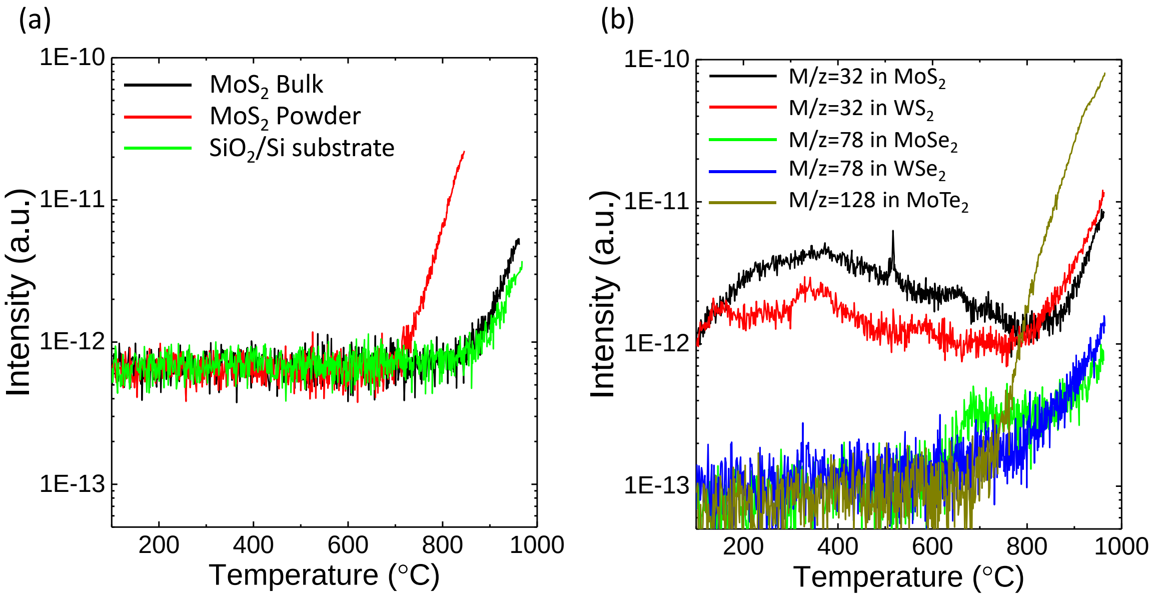

Fig. S2 (a) Comparison of TDS spectra of M/z = 32 for MoS_2 flakes and MoS_2 powder in the second annealing. (b) Comparison of TDS spectra of M/z = 32 for MoS_2 and WS_2, M/z = 78 for $MoSe_2$ and WSe_2, M/z = 128 for $MoTe_2$ in the first annealing.

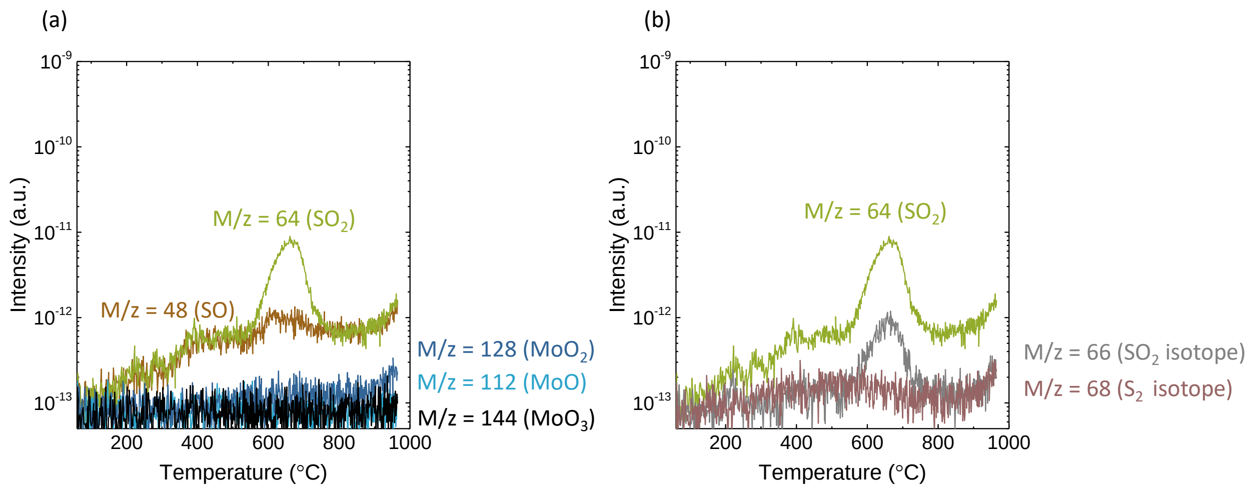

Fig. S3 (a) TDS spectra for bulk MoS_2 repeated for the detection of SO (M/z = 48) and SO_2 (M/z = 64). The Mo oxides including MoO_2 (M/z = 128), MoO (M/z = 112) and MoO_3 (M/z = 144) are included to exclude the desorption from Mo oxides. (b) TDS spectra for the identification of SO_2 desorption by monitoring the desorption signals include sulfur isotopes (M/z = 66 and M/z = 68).

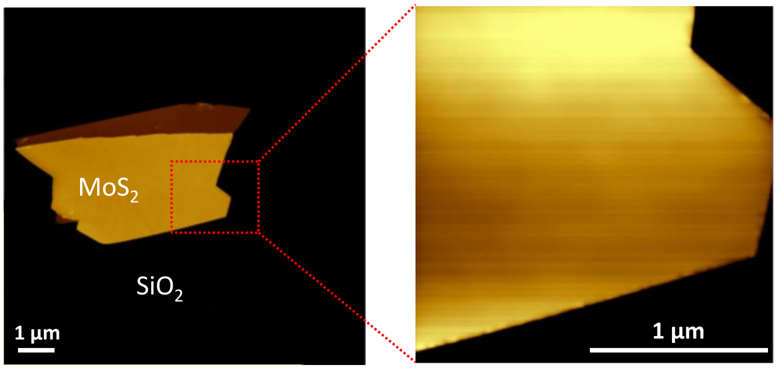

Fig. S4 AFM images for bulk MoS_2 (52 nm) annealed up to 835 °C in the TDS chamber without ALD. No defects can be observed due to the limited resolution of the AFM apparatus.

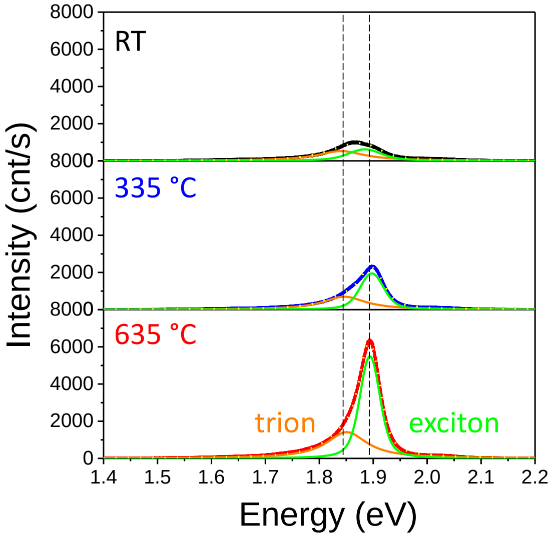

Fig. S5 Deconvolution of PL spectra at different temperatures in **Fig. 4a** to trion (orange) and exciton (green) by Voigt function.
